# Reframing Polypharmacy: Empowering Medical Students to Manage Medication Burden as a Chronic Condition

**DOI:** 10.3390/clinpract15080142

**Published:** 2025-07-31

**Authors:** Andreas Conte, Anita Sedghi, Azeem Majeed, Waseem Jerjes

**Affiliations:** 1 Faculty of Life Sciences & Medicine, King’s College London, London SE1 1UL, UK; andreas.conte@kcl.ac.uk; 2 Faculty of Medicine, Imperial College London, London SW7 2AZ, UK; anita.sedghi@nhs.net (A.S.); a.majeed@imperial.ac.uk (A.M.)

**Keywords:** polypharmacy, medical education, deprescribing, multidisciplinary collaboration, diagnostic tools, patient-centred care, medication management

## Abstract

**Aims/Background:** Polypharmacy, or the concurrent intake of five or more medications, is a significant issue in clinical practice, particularly in multimorbid elderly individuals. Despite its importance for patient safety, medical education often lacks systematic training in recognising and managing polypharmacy within the framework of patient-centred care. We investigated the impact of a structured learning intervention introducing polypharmacy as a chronic condition, assessing whether it enhances medical students’ diagnostic competence, confidence, and interprofessional collaboration. **Methods:** A prospective cohort study was conducted with 50 final-year medical students who received a three-phase educational intervention. Phase 1 was interactive workshops on the principles of polypharmacy, its dangers, and diagnostic tools. Phase 2 involved simulated patient consultations and medication review exercises with pharmacists. Phase 3 involved reflection through debriefing sessions, reflective diaries, and standardised patient feedback. Student knowledge, confidence, and attitudes towards polypharmacy management were assessed using pre- and post-intervention questionnaires. Quantitative data were analysed through paired *t*-tests, and qualitative data were analysed thematically from reflective diaries. **Results:** Students demonstrated considerable improvement after the intervention in identifying symptoms of polypharmacy, suggesting deprescribing strategies, and working in multidisciplinary teams. Confidence in prioritising polypharmacy as a primary diagnostic problem increased from 32% to 86% (*p* < 0.01), and knowledge of diagnostic tools increased from 3.1 ± 0.6 to 4.7 ± 0.3 (*p* < 0.01). Standardised patients felt communication and patient-centredness had improved, with satisfaction scores increasing from 3.5 ± 0.8 to 4.8 ± 0.4 (*p* < 0.01). Reflective diaries indicated a shift towards more holistic thinking regarding medication burden. The small sample size limits the generalisability of the results. **Conclusions:** Teaching polypharmacy as a chronic condition in medical school enhances diagnostic competence, interprofessional teamwork, and patient safety. Education is a structured way of integrating the management of polypharmacy into routine clinical practice. This model provides valuable insights for designing medical curricula. Future research must assess the impact of such training on patient outcomes and clinical decision-making in the long term.

## 1. Introduction

Polypharmacy is generally defined as the concurrent use of five or more medications and has become a critical issue in clinical practice, especially among older adults and those with multiple chronic conditions [1]. The rising prevalence of polypharmacy reflects the success of modern medicine in managing individual diseases but also creates a significant challenge: the additive effect of multiple medications on patient well-being. Beyond the pharmacological connotations, polypharmacy leads to adverse drug reactions, drug interactions, reduced quality of life, and functional impairment [2].

Polypharmacy significantly increases the risk of adverse drug reactions (ADRs) in older adults. A study involving 592 participants aged 70 and above found a cumulative ADR incidence of 26.9% over six years, with 10.9% of these reactions classified as moderate and 3.8% necessitating emergency hospital admissions. Notably, ADRs were independently associated with polypharmacy, defined as the concurrent use of five to nine drug classes (adjusted odds ratio [OR] 1.81), and major polypharmacy, defined as the use of ten or more drug classes (adjusted OR 3.33) [3].

The philosophy of medical education has always been disease-centred, where management of conditions is focused in isolation [4]. While this might be appropriate in single-disease situations, this model often creates a snowball effect of prescriptions in a multimorbid patient, wherein drugs prescribed to manage one condition inadvertently trigger symptoms that may be treated with other medications [5]. For example, a patient prescribed amlodipine for hypertension may subsequently experience ankle oedema, leading to the addition of furosemide, which then triggers electrolyte imbalance requiring potassium supplementation—thereby initiating a prescribing cascade. This becomes a vicious circle that worsens the polypharmacy burden and often leads to misdiagnosis and interventions which further complicate the patient’s condition.

Deprescription refers to the structured process of tapering, withdrawing, discontinuing, or reducing medications to manage polypharmacy effectively, improve patient outcomes, and prevent medication-related harm, especially critical in elderly patients with multiple chronic conditions. What is required, therefore, is a paradigm shift in integrating polypharmacy into the differential diagnoses of medical students to give the future clinician the necessary skill to recognise and manage medication-related problems as an integral part of patient care [6].

Reframing polypharmacy as a chronic condition encourages a more holistic, patient-centred approach to healthcare. Polypharmacy management requires a multidisciplinary approach, collaborating closely with pharmacists and other healthcare professionals [7]. In the context of an ongoing, dynamic process, medical students must learn to consider medication burden alongside other chronic conditions such as diabetes or hypertension [8,9,10]. This would engender a diagnostic mindset that underscores regular review of medications, identification of drug-induced symptoms, and prevention of adverse effects. This is in line with the broader healthcare goals of improving the patient’s safety, enhancing their quality of life, and reducing unnecessary healthcare expenditure [10,11,12].

Despite the well-documented impact of polypharmacy on patient outcomes, medical education has yet to develop a formal framework for integrating the management of polypharmacy into clinical education. Existing curricula place greater emphasis on disease-directed treatment to the detriment of comprehensive, patient-centred approaches to medication burden. Such a limited approach reinforces prescribing cascades, misdiagnosis of drug effects, and fragmented care. With the increasing prevalence of polypharmacy, particularly in the elderly and in those with multimorbidity, there is an urgent need to equip future clinicians with the competencies to recognise and manage medication burden proactively.

In terms of education around polypharmacy, various models have been employed to enhance the competency of healthcare professionals [13]. For instance, one study assessed the impact of a polypharmacy Action Learning Set (ALS) instrument upon the confidence and capability of healthcare practitioners managing polypharmacy and found the value of structured educational interventions in promoting appropriate medication use. Similarly, a study with pharmacy students identified that education around deprescribing within the curriculum had a positive influence upon their confidence and preparedness to practice deprescribing interventions, highlighting the value of including such content within educational courses [14]. In addition, simulation-based exercises such as the Jellybean Polypharmacy Simulation Exercise (JPSE) have also been used to enhance empathy and appreciation within pharmacy students for the complexity of polypharmacy and suggest the value of experiential approaches as a complement to conventional didactic teaching [15].

The objective of this study was to address the insufficient emphasis on medication management in existing medical curricula. This would be achieved by ascertaining whether a systematic, three-phase educational intervention would enhance medical students’ identification and management of polypharmacy. The study particularly aimed to (1) increase students’ confidence in prioritising the symptoms of polypharmacy as a primary diagnostic issue, (2) enhance knowledge of deprescribing processes and diagnostic tools, (3) build multidisciplinary collaboration skills, particularly in working with pharmacists, and (4) enhance communication skills in the consultation of polypharmacy patients. It was predicted that students exposed to the intervention would exhibit significant improvement from their baseline level in these parameters, as ascertained using pre- and post-intervention surveys, standardised patient feedback, and reflective diaries.

## 2. Participants and Methods

### 2.1. Study Design

This study was a prospective cohort study evaluating the impact of a structured educational intervention on the ability of medical students to recognise and manage polypharmacy. The intervention was founded on a three-phase approach, comprising interactive workshops, simulated patient consultations, and reflective learning. Participants were assessed at baseline and post-intervention using a combination of quantitative questionnaires, standardised patient evaluations, and qualitative reflective journal analysis Appendix A and Appendix B. The study adhered to best evidence medical education research principles, enabling a rigorous assessment of student learning outcomes in polypharmacy management.

### 2.2. Setting

Final-year undergraduate medical students were given a structured educational intervention in polypharmacy management. The intervention was conducted in classroom-based learning environments, simulation labs, and small-group clinical skills workshops. The sessions were conducted by senior clinical teachers, who were general practitioners, pharmacists, and geriatricians. Data collection was between [March 2022–March 2024], with student recruitment, educational sessions, and assessments all occurring within a defined academic term.

### 2.3. Participants

Final-year medical students were invited to participate in this study. The inclusion criteria were that the students had to have completed at least two clinical rotations in primary care, internal medicine, or geriatrics to have been exposed to polypharmacy-related clinical scenarios. Students were recruited through targeted email invitations and medical school administrator announcements. Participation was voluntary, and informed consent was obtained from all students before enrolment. Students were incentivised through certification of participation, enhancement of their clinical portfolios, and additional exposure to polypharmacy management.

Eligible students consented to take part in all three phases of the educational intervention and undertake pre- and post-intervention measures, including surveys, standardised patient encounters, and reflective journaling. Exclusion criteria included prior training in deprescribing or polypharmacy management, inability to complete the full intervention, or withdrawal of consent at any time.

### 2.4. Variables and Quantitative Measures

The primary variables assessed were students’ knowledge, confidence, diagnostic capacity, multidisciplinary collaboration, and communication skills in polypharmacy management. The educational intervention (independent variable) consisted of interactive workshops, simulated patient consultations, and reflective learning. Dependent variables were measured as pre- and post-intervention changes assessed through questionnaires, standardised patient feedback, and reflective journals.

Confidence in identifying polypharmacy-related symptoms was measured using a 5-point Likert scale from 1 (“Not at all confident”) to 5 (“Very confident”). Knowledge of deprescribing practices and diagnostic tools was evaluated using a multiple-choice quiz with scores ranging from 0 to 5. Communication quality, including clarity, patient engagement, and shared decision-making, was assessed through standardised patient satisfaction ratings on a 5-point scale.

Secondary quantitative outcomes included the number of drug–drug interactions identified per patient record, the proportion of students accurately identifying unnecessary medications, listing polypharmacy as a top diagnostic consideration, and involving pharmacists in deprescribing decisions. Secondary qualitative outcomes included students’ ability to collaboratively identify drug-induced symptoms and recommend deprescribing strategies, assessed through qualitative thematic analysis of reflective diaries and structured multidisciplinary consultations.

### 2.5. Data Sources and Measurement

Quantitative and qualitative data were collected from multiple sources to ascertain the impact of the educational intervention. Pre- and post-intervention questionnaires used Likert-scale questions to measure students’ confidence in recognising polypharmacy-related symptoms, knowledge of deprescribing approaches, and comfort in communicating with multidisciplinary teams.

The questionnaire used a 5-point Likert scale [16], where each item was scored from 1 (low confidence/strongly disagree) to 5 (high confidence/strongly agree). The scale included items covering different aspects of polypharmacy management, with overall scores calculated as the mean of individual item scores. Higher scores indicated greater confidence and competence in the assessed domains.

Standardised patients provided structured feedback on students’ communication skills and effectiveness in communicating about medication management. Objective performance metrics, such as the number of drug–drug interactions correctly identified and deprescribing approaches recommended, were also assessed during medication review exercises.

Qualitative data were collected from reflective diaries and debriefing session transcripts. These were analysed and independently coded by two researchers to examine students’ attitude change towards polypharmacy management. These reflections also provided insight into students’ diagnostic reasoning, attitude towards patient-centred care, and their ability to apply knowledge to clinical practice. All data were de-identified and anonymously collected for analysis.

A phased analysis approach was used to assess the sequential and cumulative impact of each intervention phase. Outcomes were specifically analysed at three distinct points: after theoretical workshops (Phase 1), after practical training (Phase 2), and following reflective learning (Phase 3).

### 2.6. Interventional Methods

The project followed a structured, three-phase methodology of embedding students into the concept of polypharmacy as a chronic condition and equipping them with practical tools for its effective management (Table 1). Representative samples of educational materials from each intervention phase are presented in Appendix C.

The first phase introduced polypharmacy principles, risks including adverse drug reactions, and prescribing cascades, using a case-based and technology-integrated approach.

Case-based learning allowed students to analyse real-life scenarios of how polypharmacy was affecting patient outcomes, such as cognitive impairment due to a prescribing cascade. Diagnostic strategies were introduced that emphasised the identification of symptoms related to polypharmacy, such as fatigue, dizziness, and gastrointestinal complaints, and the differentiation of these from disease progression. Students were also trained in the use of Electronic Health Records (EHRs) and decision-support systems (CDSS) in the identification of drug interactions and flagging medications requiring review. The pre-survey consisted of a combination of Likert-scale and open-ended questions to gather the initial views of students related to perceived challenges in clinical practice and base knowledge and level of comfort regarding polypharmacy recognition and management (Appendix A).

In the second phase, students applied knowledge practically through simulated patient interactions and medication review exercises, focusing on identifying drug-induced symptoms and proposing deprescribing strategies collaboratively.

In the third phase, students engaged in facilitated debriefing and reflective journaling, focusing on integrating polypharmacy management insights and strategies into future practice.

Quantitative data from pre- and post-workshop survey responses measured students’ knowledge, confidence, and skills pre- and post-workshop. Scores obtained from Likert-scale items were summarised as means with standard deviations. Statistical significance was determined using paired *t*-tests. Qualitative data-reflective journals, debriefing session transcripts, and feedback from standardised patients-were thematically analysed to highlight key insights, challenges, and proposed strategies for managing polypharmacy (Appendix B).

### 2.7. Bias

Selection bias was limited by recruitment of a diverse group of final-year medical students, with some variation in clinical exposure and experience in polypharmacy management. Recruitment was on a voluntary basis, and advertising material emphasised that the research was an educational project rather than an assessment of student performance.

To minimise response bias, pre- and post-intervention surveys were anonymised to facilitate candid self-reflection free from academic repercussion. Observer bias in standardised patient evaluations was minimised by using structured feedback forms with pre-determined criteria for assessing communication and patient engagement. Lastly, confirmation bias in qualitative analysis was reduced by having multiple researchers code themes independently to encourage objective interpretation of reflective journal data.

### 2.8. Study Size

The sample size was determined based on a prior power calculation to ensure adequate statistical power for detecting meaningful changes in students’ knowledge, confidence, and diagnostic capabilities. A power analysis was conducted using an expected effect size of 0.8, a significance level (α) of 0.05, and a power of 80%, indicating that a minimum of 50 participants would be required to detect statistically significant pre- and post-intervention differences.

To assess the impact of the intervention, Number Needed to Treat (NNT) was estimated based on the proportion of students demonstrating a significant improvement in identifying polypharmacy-related symptoms and formulating deprescribing strategies. Given that baseline confidence in identifying polypharmacy as a primary diagnostic concern was 32% and increased to 86% post-intervention (*p* < 0.01), the NNT for achieving a meaningful improvement in diagnostic confidence was 1.9 (1/absolute risk reduction of 54%). Similar calculations were applied to deprescribing strategy formulation and multidisciplinary collaboration, reinforcing the effectiveness of the intervention.

### 2.9. Statistical Methods

Statistical analysis was primarily performed using paired *t*-tests to compare differences before and after the intervention for continuous variables, such as student confidence in recognising symptoms associated with polypharmacy, their knowledge about deprescribing practices, and ratings provided by standardised patients. To verify assumptions for the use of parametric tests, quantitative variables underwent normality testing using the Shapiro–Wilk test. In cases where data met the normality criteria, paired *t*-tests were used, with statistical significance set at a *p*-value below 0.05.

Categorical outcomes, including the proportion of students who identified polypharmacy as a critical diagnostic issue, those who recommended deprescribing interventions, and those effectively engaging in multidisciplinary teamwork, were compared pre- and post-intervention using McNemar’s test for paired nominal data.

For continuous or ordinal paired data that did not fulfil normal distribution assumptions, such as Likert-scale assessments of confidence and knowledge, the Wilcoxon signed-rank test was applied. Selection of statistical tests was determined by considering the nature of the variables and their underlying distribution patterns.

Potential confounding variables were addressed by stratifying the students based on previous clinical experiences, such as rotations in geriatrics, internal medicine, or primary care. Subgroup analyses were conducted to compare outcomes between students with and without prior exposure to polypharmacy management scenarios, using independent *t*-tests for continuous variables and chi-square tests for categorical variables.

Due to minimal missing data, analyses were conducted using a complete-case approach, justified by the fact that students completing post-intervention evaluations had consistently participated in pre-intervention measures. Sensitivity analyses were carried out to ensure robustness of findings when excluding data from any participants who missed portions of the intervention. Since all participants completed both pre- and post-intervention assessments, no further adjustments for attrition were necessary.

### 2.10. Ethical Considerations

This study focused on educational and training interventions, specifically designed to enhance medical students’ understanding and practical management of polypharmacy. Given the educational and observational nature of the research, involving no interventions or changes to actual patient care or clinical decisions, formal ethical approval was not required as per institutional guidelines.

All participants provided informed consent prior to involvement in the study. Participation was completely voluntary, and students were fully briefed about the study objectives, methodology, and potential outcomes beforehand. Additionally, standardised patients involved in simulation exercises also provided explicit informed consent for their participation.

The research adhered fully to the ethical principles outlined in the Declaration of Helsinki. Confidentiality was strictly maintained, with each participant being assigned a unique, anonymised identifier to safeguard privacy. All collected data—including questionnaire responses, reflective journal entries, and simulation session feedback—were securely stored in line with the institution’s data protection policies to ensure confidentiality and anonymity.

Future iterations of this educational initiative will expand the involvement of actual patients, integrating elements such as patient-facilitated feedback or patient testimonial-based discussions. These enhancements aim to further enrich the educational experience, deepen student empathy, and strengthen the focus on patient-centred care and effective polypharmacy management.

## 3. Results

### 3.1. Participants

**Figure 1:**
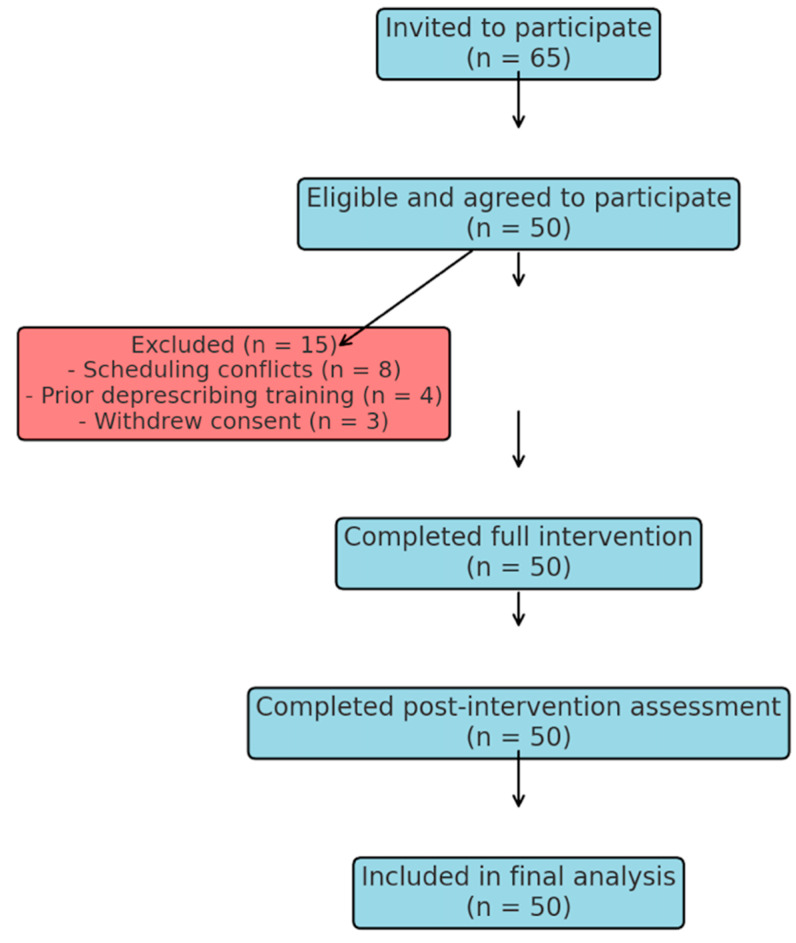
Flowchart illustrating the recruitment, eligibility assessment, exclusion reasons, intervention completion, and final analysis of medical students participating in the study.

A total of 65 last-year medical students were contacted to participate in the study, and 50 students (76.9%) fulfilled the inclusion criteria and were available to participate. The exclusion reasons (n = 15, 23.1%) included primarily scheduling (n = 8), prior specialised education in deprescribing (n = 4), and withdrawal of consent before receiving the intervention (n = 3). The 50 participants who were included (100%) attended until the end of the intervention and post-study assessment, and no loss to follow-up occurred (Figure 1). Participants included 30 females (60%) and 20 males (40%), with ages ranging from 22 to 26 years (mean 24.2); ethnicities were 52% Asian, 24% White, 14% Black, and 10% Mixed/Other backgrounds.

### 3.2. Impact on Knowledge and Confidence

Analyses of pre- and post-reflection questionnaires indicated significant improvements in knowledge and confidence for the students regarding managing polypharmacy. At baseline, the average confidence score among all 50 participants in identifying polypharmacy-related symptoms as a primary diagnostic concern was 2.9 ± 0.7 (Table 2). Following the intervention, this increased to 4.5 ± 0.4 (*p* < 0.01), demonstrating a statistically significant improvement. Additionally, the proportion of students rating their confidence as “high” (≥4 on the scale) increased from 32% pre-intervention to 86% post-intervention. Knowledge of diagnostic strategies to manage polypharmacy-related problems also increased substantially. At baseline, the average knowledge score for all 50 participants on diagnostic tools for identifying drug–drug interactions and prescribing cascades was 3.1 ± 0.6. Post-workshop, this increased to 4.7 ± 0.3 (*p* < 0.01). Additionally, the proportion of students who rated their confidence as “high” (≥4 on the scale) increased from 84% pre-intervention to 94% post-intervention. At baseline, the average confidence score for all 50 participants in multidisciplinary collaboration was 3.2 ± 0.7. Following the intervention, this increased to 4.6 ± 0.4 (*p* < 0.01). Additionally, the proportion of students who rated their confidence as “high” (≥4 on the scale) increased from 32% pre-workshop to 86% post-workshop.

### 3.3. Phased Analysis of Intervention Impact

To further evaluate the effectiveness of the intervention, student performance was analysed across three distinct phases: after theoretical learning (Phase 1: workshops), after practical training (Phase 2: simulated patient interactions and medication reviews), and after reflection (Phase 3: debriefing and reflective journals).

Confidence in Identifying Polypharmacy-Related Symptoms: After the workshops, confidence improved from a baseline of 2.9 ± 0.7 to 3.8 ± 0.5 (*p* < 0.05). Following practical training, confidence further increased to 4.2 ± 0.4 (*p* < 0.01), and after reflection, final scores reached 4.5 ± 0.4 (*p* < 0.01), indicating cumulative improvement.

Knowledge of Diagnostic Tools: Awareness of deprescribing tools increased from 3.1 ± 0.6 at baseline to 3.9 ± 0.5 (*p* < 0.05) after workshops. Post-practical phase, scores rose to 4.4 ± 0.3, with a final post-reflection score of 4.7 ± 0.3 (*p* < 0.01).

Confidence in Multidisciplinary Collaboration: After Phase 1, awareness of the role of pharmacists in medication review increased from 3.2 ± 0.7 to 3.7 ± 0.5. By Phase 2, this improved further (4.2 ± 0.4), reaching 4.6 ± 0.4 post-reflection (*p* < 0.01).

Performance in Simulated Patient Interactions: Before the intervention, only 38% of students correctly identified polypharmacy-related symptoms. This increased to 64% after theoretical learning, 79% after practical training, and 88% after reflective discussions (*p* < 0.01). Similarly, the proportion of students able to propose deprescribing strategies increased from 34% pre-intervention to 58% after workshops, 74% after practical exercises, and 90% post-reflection.

To complement these outcomes, additional domains such as communication skills, diagnostic ability, and drug interaction identification were also assessed across phases. These results, presented in Table 3, show consistent improvement across all competencies with statistically significant gains, particularly following the reflective phase.

In parallel, Table 4 shows the proportion of students achieving specific behavioural competencies across the intervention. Statistically significant improvements were observed across all domains, including the ability to engage patients in discussions and collaborate effectively with multidisciplinary teams. These data reinforce the intervention’s broad impact on both technical knowledge and patient-centred communication.

### 3.4. Impact of Clinical Experience on Learning Outcomes

- Baseline confidence scores:
  ○ Students with prior geriatrics exposure: 3.1 ± 0.6
  ○ Students without prior exposure: 2.7 ± 0.7 (*p* = 0.07, nonsignificant)
- Post-intervention confidence:
  ○ With geriatrics exposure: 4.6 ± 0.3
  ○ Without geriatrics exposure: 4.4 ± 0.4 (*p* = 0.04)
- Knowledge of deprescribing tools:
  ○ With prior exposure: 3.4 ± 0.5 → 4.8 ± 0.3
  ○ Without exposure: 2.9 ± 0.6 → 4.5 ± 0.3 (*p* < 0.01)

#### Nonsignificant Findings

- Confidence in differentiating adverse drug reactions improved from 3.5 ± 0.7 to 3.9 ± 0.6 (*p* = 0.08)
- Understanding of pharmacokinetic changes improved from 3.2 ± 0.6 to 3.5 ± 0.5 (*p* = 0.09)

### 3.5. Performance in Simulated Patient Interactions

During the role-playing scenarios, there was a notable improvement in students’ ability to identify and address polypharmacy-related issues. Prior to the intervention, only 38% of students correctly identified symptoms such as fatigue, confusion, or dizziness as potentially drug-induced. This increased significantly to 88% post-intervention, enhancing the specificity of their diagnostic and management approaches. Additionally, the percentage of students who developed actionable deprescribing strategies rose from 34% before training to 78% after training.

Standardised patients reported increased empathy and overall engagement from the students. They felt that students were highly attentive to their questions and concerns regarding medications. They noted significant improvements in students’ communication clarity, particularly in explaining medication adjustments and involving patients actively in shared decision-making. Standardised patient ratings for students’ communication as “excellent” increased notably from 40% before the workshops to 84% afterward, reflecting improved patient-centred care.

### 3.6. Insights from Medication Review Exercises

**Figure 2:**
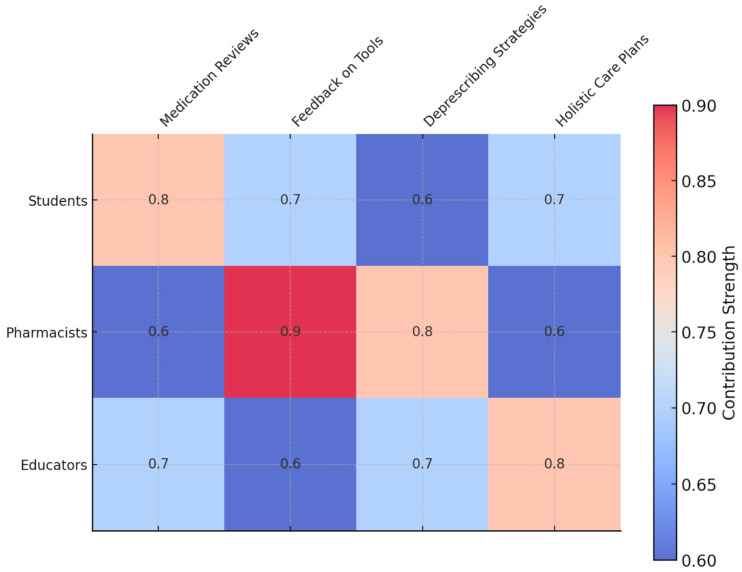
Matrix diagram: contribution strengths of roles to outcomes in polypharmacy management. This matrix visualises the relative contributions of students, pharmacists, and educators to key outcomes, such as medication reviews, diagnostic feedback, and holistic care plans. The colour intensity highlights the strength of each contribution, with pharmacists showing the highest input in feedback on tools and educators leading in holistic care planning. This diagram emphasises the collaborative nature of the intervention, showcasing how each role uniquely impacts the outcomes of polypharmacy management training.

These small group medication review exercises allowed the students to practice their critical appraisal of complex medication regimens. Analysing anonymised patient records highlighted repeating issues including drug–drug interaction, inappropriate medication use, and cascading. Students identified an average 2.4 drug–drug interactions per patient record pre-training increased to 4.7 drug–drug interactions per record post-training (Table 2). Feedback from multidisciplinary discussions also indicated that students felt increasingly comfortable in consulting pharmacists and using their insights to inform final recommendations (Figure 2).

### 3.7. Reflective Insights

Reflective journals and debriefing sessions provided rich qualitative insights into the students’ experiences (Table 5). One theme that strongly emerged was an appreciation of the need to consider polypharmacy as a major diagnostic consideration. For most students, their surprise at the degree to which medications may simulate or exacerbate disease processes was a new insight. One student commented, “I never would have thought to consider polypharmacy as a chief cause for symptoms such as fatigue or dizziness. This experience completely changed the way I approach patient presentations.” Students also reflected on the value of multidisciplinary collaboration. As one participant noted, “Working with the pharmacist helped me see the big picture of the patient’s medication regimen. Their input was invaluable in making safer, more effective recommendations.” These reflections underlined the importance of teamwork and showed the transformation that had taken place within these students with regard to diagnostic and communication skills.

## 4. Discussion

### 4.1. Key Results

This intervention enhanced diagnostic skill, confidence, and teamwork in an interprofessional approach by redefining polypharmacy as a chronic disease. The students exhibited enhanced recognition of polypharmacy, increased ability to propose deprescribing, and enhanced interaction between pharmacists and simulated patients in medication review.

The role of prior clinical experience in learning outcomes was evident in this study. Students who had prior exposure to geriatrics or internal medicine exhibited slightly higher baseline confidence and knowledge scores, but the intervention significantly improved outcomes for all participants. These differences likely reflect greater familiarity with polypharmacy-related challenges rather than differences in the intervention’s effectiveness. Future studies could explore whether targeted pre-course preparation enhances outcomes further for those with less prior clinical exposure. Table 6 presents representative clinical scenarios illustrating how students practically applied their knowledge, reasoning, and deprescribing strategies gained from the structured intervention, highlighting their improved competence in managing polypharmacy among elderly patients with multiple comorbidities.

Some aspects of polypharmacy management, such as differentiating adverse drug effects from underlying disease and pharmacokinetic considerations, showed limited improvement despite the intervention. This may reflect the intrinsic complexity of these topics, requiring longer-term reinforcement through clinical exposure rather than short-term education. Additionally, some students may have had baseline familiarity with these concepts, leading to a smaller detectable knowledge gain. Future iterations of the intervention could incorporate case-based learning and real-world patient interactions to enhance understanding of these nuanced areas.

### 4.2. Limitations

Despite the significant improvement in students’ understanding and organisation of polypharmacy, there are various limitations in the current study. The lack of a control group is one limitation, and therefore, whether improvement observed is solely attributed to the intervention or is influenced by extraneous factors such as concurrent clinical placements cannot be gauged. Future studies need to use a randomised controlled trial to strengthen causal inferences.

Another limitation is response bias in students’ self-assessed confidence and knowledge. Objective measurement by standardised patient feedback and medication review exercises was included, but students may have exaggerated their improvement in learning by means of social desirability bias or the Hawthorne effect, in which participants modify their behaviour by mere observation. Anonymised follow-up testing may alleviate such an effect in future studies.

Additionally, the population sample (n = 50), though power-calculated, included only one university cohort in London, and generalisability to institutions and health settings, in general, is thus compromised. Further multicentre studies to evaluate reproducibility in different curricula and student populations are necessary.

The study also relied on simulated interactions with patients, which are good for structured learning but insufficient to capture clinical decision-making in daily life. Longitudinal follow-up in real patients in future interventions is required to verify whether skill acquired through the intervention is transferred to regular clinical practices. In subsequent studies, we plan to implement delayed follow-up assessments at 6-month and 12-month intervals post-intervention, enabling evaluation of the sustained impact of the training on students’ clinical decision-making skills, confidence, and knowledge retention in managing polypharmacy effectively in clinical practice.

Given the small cohort size and lack of a control group, we acknowledge potential limitations in definitively attributing improvements solely to our intervention; thus, we performed sensitivity analyses and interpret the outcomes cautiously, recommending that future studies employ larger, controlled designs to robustly assess intervention effectiveness.

Finally, while the research did identify benefits to multidisciplinary collaboration, collaboration between practicing pharmacists and healthcare teams remained relegated to structured learning exercises. Expansion of opportunities for experiential interprofessional collaboration may better prepare students to include such skill in clinical settings.

### 4.3. Interpretation

The findings of this study suggest that reframing polypharmacy as a chronic condition can enhance medical students’ diagnostic acumen, confidence, and interdisciplinary collaboration. The significant improvements in recognising polypharmacy-related symptoms, proposing deprescribing strategies, and engaging in shared decision-making align with previous research advocating for patient-centred approaches to polypharmacy management [10,17,18]. These results reinforce the notion that medical education should shift away from disease-centred models toward more holistic medication management frameworks, particularly in populations with multimorbidity [19,20,21].

However, caution is warranted in such interpretation in light of limitations in the study, including no control group, simulated interactions, and response bias. The improvement in confidence and knowledge, while found to be statistically significant, is questionable in their clinical impact in the longer term. Longitudinal studies following up on students’ application and maintenance of such skill in clinical settings is necessary to evaluate whether students retain and effectively use them.

The intervention’s focus on experiential learning through workshops and simulation aligns with educational models emphasising active learning and reflection [20,22]. Similar studies have shown that integrating interprofessional collaboration into medical education enhances students’ ability to work with pharmacists and other healthcare professionals [23,24]. However, the limited real-world pharmacist involvement in this study suggests that future iterations should expand practical multidisciplinary exposure beyond structured educational settings [25,26].

Additionally, while the findings indicate strong short-term gains, polypharmacy remains a complex, systemic issue requiring ongoing clinical reinforcement. As suggested in prior research [27,28], incorporating EHR-based tools and CDSS into training may help sustain diagnostic and deprescribing skills over time. The integration of emerging technologies, such as pharmacogenomics, may further improve personalised medication management [20,29,30]. In summary, this study presents encouraging evidence for better, patient-centric prescribing practices through reframing polypharmacy as a chronic disease in education. In order to take such evidence to long-lasting clinical benefits, future studies should evaluate longer effects, larger institutional adoption, and implementation of innovative technology tools in education on polypharmacy.

While improved confidence and knowledge post-training are expected outcomes, the critical contribution of this study lies in its unique, structured approach, enhancing medical education and patient safety.

### 4.4. Generalisability

The simulated patient interactions on which the trial is focused, while convenient for standardisation, may not fully capture clinical practice in everyday life. Feasibility and impact of the intervention may be influenced by variations in prescribing culture, health system resources, and influence of pharmacists between health settings and geographic regions. An extension to multiple institutions and to real-life patient interactions may improve the external validity of evidence. Despite these limitations, the core principles of this educational model—experiential learning, diagnostic reframing, and multidisciplinary collaboration—align with broader trends in medical education. Similar studies have shown that early integration of polypharmacy management into training improves clinical decision-making and patient outcomes [17,19,21]. Future studies should explore how this intervention can be adapted for different training environments, including postgraduate medical education, nursing, and pharmacy curricula, to enhance its generalisability across healthcare professions. Future research will focus on validating this structured educational intervention across various healthcare disciplines, such as pharmacy, nursing, and other allied health professions, to comprehensively evaluate its effectiveness, practicality, and broader applicability within interprofessional education and practice contexts.

### 4.5. Bridging Education and Policy in Managing Polypharmacy

While this evidence demonstrates the feasibility of reframing polypharmacy as a chronic disease in medicine education, its health system and policy impact is in greater need of assessment. Polypharmacy is no only an education issue for medicine but is an endemic health phenomenon influenced by prescribing behaviour, health system demands, and regulatory requirements. The incorporation of polypharmacy management in education models, postgraduate curricula, and ongoing professional development (CPD) programmes may standardise optimal practices between health disciplines.

A key next step is ensuring that policy frameworks align with educational efforts to support safer prescribing. Existing national guidelines, such as the STOPP/START criteria and the Beers Criteria, serve as essential reference points but are often underutilised in routine clinical practice [21,28]. Medical education must bridge this gap by ensuring that students and early-career clinicians are proficient in applying these guidelines in real-world settings. Additionally, interprofessional collaboration should be embedded beyond simulated exercises, with structured pharmacist-led deprescribing initiatives incorporated into both undergraduate and postgraduate medical training [13,24]. We intend to adapt future iterations of this intervention by formally integrating interprofessional training components, creating structured opportunities for medical, pharmacy, nursing, and other healthcare students to collaboratively learn deprescribing strategies and medication review skills, thus fostering a more cohesive, interdisciplinary approach to managing polypharmacy.

Furthermore, healthcare system constraints—such as time pressures, resource limitations, and fragmented care pathways—can hinder effective polypharmacy management. Policymakers and healthcare institutions should prioritise integrating clinical decision support systems (CDSS) within electronic health records (EHRs) to facilitate deprescribing decisions in a time-efficient manner [27]. Future research should assess how scaling educational interventions to a national or global level can improve patient safety and reduce inappropriate prescribing trends, ultimately enhancing patient outcomes.

By bridging health policy and education in medicine, such evidence by this study holds the potential to contribute to an expanded, system-level improvement in addressing polypharmacy, such that education interventions have effects on health system and clinical improvement.

## 5. Conclusions

This study demonstrates how education on polypharmacy in medical education augments diagnostic ability, patient protection, and provision of compassionate, patient-centric care. Redefined, polypharmacy as a chronic disease facilitates diagnostic thinking, enabling students to recognise and approach one of today’s most pressing health issues. The evidence highlights the need for collaborative, multifaceted education, reflection on learning, and use of technology in providing future clinicians with confidence and ability to manage the complexities of polypharmacy. For sustainability, such education strategies should be integrated into medical curricula, postgraduate education, and ongoing professional education to strengthen system-level prescribing and deprescribing.

## Figures and Tables

**Table 1:** Summary of educational phases: activities, objectives, and outcomes in polypharmacy training.

Phase	Key Activities	Learning Objectives	Outcomes Measured
Phase 1: Interactive workshops	- Introduction to polypharmacy: Definition, prevalence, and associated risks, including adverse drug reactions and prescribing cascades. - Case-based learning: Real-world case studies showcasing the impact of polypharmacy on patient outcomes. - Diagnostic tools and strategies: Training in recognising polypharmacy-related symptoms and differentiating these from disease progression. - Technology integration: Hands-on practice using EHRs and CDSS to identify potential drug interactions and medications requiring review.	- Understand the prevalence and implications of polypharmacy in clinical practice. - Develop foundational knowledge of diagnostic tools and strategies for managing polypharmacy. - Build familiarity with technology tools to enhance medication safety.	- Baseline knowledge and confidence scores from pre-workshop surveys. - Student understanding of polypharmacy risks and diagnostic approaches.
Phase 2: Practical application	- Simulated patient interactions: Role-playing exercises with standardised patients presenting symptoms linked to polypharmacy (e.g., fatigue, confusion, dizziness). - Medication review exercises: Small group activities reviewing anonymised patient records to identify and address issues such as drug–drug interactions and redundant medications. - Collaborative learning: Multidisciplinary discussions with pharmacists and clinical educators focusing on deprescribing strategies and holistic patient care. - Development of action plans: Formulation of patient-specific recommendations for reducing medication burden while ensuring therapeutic efficacy.	- Apply diagnostic and management skills in identifying polypharmacy-related issues. - Develop communication skills for discussing deprescribing and medication reviews with patients and caregivers. - Foster a collaborative, multidisciplinary approach to polypharmacy management.	- Performance in simulated patient interactions as rated by standardised patients. - Success in identifying drug-related problems during medication review exercises. - Multidisciplinary team feedback on student contributions and proposed interventions.
Phase 3: Reflection and evaluation	- Debriefing sessions: Facilitated group discussions to share insights, challenges, and strategies for integrating polypharmacy management into practice. - Reflective journals: Students documented their experiences, addressing themes such as diagnostic challenges, medication review strategies, and the integration of polypharmacy management into patient care. - Post-workshop surveys: Comparative analysis of pre- and post-workshop responses to assess changes in knowledge, confidence, and attitudes. - Standardised patient feedback: Evaluation of students’ communication and diagnostic performance during role-playing scenarios.	- Reflect on the practical challenges and successes of managing polypharmacy. - Consolidate learning by synthesising insights gained from the workshops and practical exercises. - Identify strategies for applying polypharmacy management skills in future clinical practice.	- Changes in confidence and knowledge scores from post-workshop surveys. - Thematic analysis of reflective journals and debriefing session transcripts. - Standardised patient feedback on communication and diagnostic skills.

**Table 2:** Quantitative outcomes of polypharmacy management training: pre- and post-intervention metrics.

Metric	Pre-Training Score (Mean ± SD)/%	Post-Training Score (Mean ± SD)/%	Change	Key Observations
Confidence in identifying polypharmacy-related symptoms	4.7 ± 0.7	4.5 ± 0.4	+1.6 points (*p* < 0.01)	Students demonstrated significant improvement in recognising symptoms like fatigue, dizziness, and confusion as potential drug effects.
Knowledge of diagnostic tools and strategies	3.1 ± 0.6	4.7 ± 0.3	+1.6 points (*p* < 0.01)	Marked increase in familiarity with tools for identifying prescribing cascades and drug–drug interactions.
Awareness of multidisciplinary collaboration	3.2 ± 0.7	4.6 ± 0.4	+1.4 points (*p* < 0.01)	Students gained a deeper understanding of the importance of pharmacist collaboration in managing polypharmacy.
Confidence in proposing deprescribing strategies	e.g., 3.0 ± 0.6	4.3 ± 0.5	+1.3 points (*p* < 0.01)	Marked improvement in confidence and ability to propose safe medication reduction strategies
Standardised patient satisfaction ratings → Communication skills	3.5 ± 0.8	4.8 ± 0.4	+1.3 points (*p* < 0.01)	Patients reported greater satisfaction with students’ ability to communicate effectively and address medication concerns.
Identification of drug–drug interactions per patient record	2.4 ± 0.6	4.7 ± 0.5	+2.3 points (*p* < 0.01)	Students significantly improved in identifying drug–drug interactions during medication review exercises.
Ability to propose deprescribing strategies (%)	34%	78%	+44%	Significant improvement in students’ ability to identify and recommend discontinuing unnecessary medications.
Students who recognised polypharmacy as a primary diagnostic consideration (%)	32%	86%	+54%	Increased awareness of polypharmacy as a central factor in patient presentations.
Students confident in engaging patients in medication discussions (%)	40%	84%	+44%	Enhanced ability to involve patients in shared decision-making around deprescribing.
Students confident in using diagnostic tools for drug–drug interactions (%)	84%	94%	+10%	Improved ability to apply tools for deprescribing strategies.
Students confident in multidisciplinary collaboration (%)	32%	86%	+54%	Stronger recognition of team-based approaches in polypharmacy.
Recognition of polypharmacy-related symptoms (%)	38%	88%	+50%	Greater awareness of symptoms attributable to medication effects.

**Table 3:** Phased analysis of continuous outcomes (Mean ± SD, n = 50).

Domain	Post-Workshop (Mean ± SD)	Post-Practical (Mean ± SD)	Post-Reflection (Mean ± SD)	t-Value (Final vs. Baseline)	*p*-Value
Confidence in identifying symptoms	3.8 ± 0.5	4.2 ± 0.4	4.5 ± 0.4	9.2	Post-workshop < 0.05; Post-practical and Post-reflection <0.01
Knowledge of diagnostic tools	3.9 ± 0.5	4.4 ± 0.3	4.7 ± 0.3	11.8	<0.01
Multidisciplinary collaboration	3.7 ± 0.5	4.2 ± 0.4	4.6 ± 0.4	10.3	<0.01
Confidence in proposing deprescribing	3.6 ± 0.6	4.0 ± 0.5	4.3 ± 0.5	8.1	<0.01
Communication skills	3.9 ± 0.6	4.3 ± 0.5	4.8 ± 0.4	8.6	<0.01
Identification of drug–drug interactions	3.5 ± 0.6	4.2 ± 0.5	4.7 ± 0.5	10.5	<0.01

**Table 4:** Phased analysis of categorical outcomes (% of students achieving outcome, n = 50).

Outcome	Post-Workshop (%)	Post-Practical (%)	Post-Reflection (%)	McNemar *p*-Value (Final vs. Baseline)
Ability to propose deprescribing	58	74	90	<0.01
Recognition of polypharmacy as a diagnostic issue	64	79	88	<0.01
Confidence in engaging patients	60	76	84	<0.01
Confidence in using diagnostic tools	88	92	94	<0.01
Confidence in multidisciplinary collaboration	72	81	86	<0.01
Recognition of polypharmacy-related symptoms	64	79	88	<0.01

**Table 5:** Thematic analysis of reflective journals: insights and implications for polypharmacy education.

Theme	Description	Illustrative Quotes	Key Implications
Recognition of Polypharmacy-Related Symptoms	Students increasingly identified drug-induced symptoms as distinct from disease progression.	“I never realised how much medications could mimic symptoms of disease. This training made me rethink my approach.”	Reinforces the need for polypharmacy to be a routine part of differential diagnosis in clinical practice.
Proactive Deprescribing	Students proposed deprescribing strategies to alleviate medication burden and improve patient outcomes.	“Reducing unnecessary medications felt like I was giving the patient their life back.”	Highlights the value of deprescribing as a core skill for improving patient quality of life.
Collaborative Problem-Solving	Students emphasised the importance of working with pharmacists and other team members in managing polypharmacy.	“The pharmacist provided insights I hadn’t considered, which made our plan more comprehensive.”	Demonstrates the value of interprofessional collaboration in addressing complex medication regimens.
Enhanced Communication Skills	Standardised patients noted improvements in students’ ability to explain medication-related issues clearly and empathetically.	“The student explained why reducing my medications could help and made me feel part of the decision.”	Empathy and clear communication are essential for patient-centred polypharmacy management.
Diagnostic Growth	Students developed a mindset that positioned polypharmacy as a primary consideration in patient assessments.	“Polypharmacy is no longer just a side note—it’s a key part of my diagnostic process now.”	Encourages future clinicians to routinely include polypharmacy in their diagnostic frameworks.
Realising Systemic Impacts	Students recognised how systemic factors, such as prescribing cascades, contribute to polypharmacy challenges.	“It’s clear that many issues stem from a fragmented healthcare approach to medications.”	Promotes a systems-based perspective to address polypharmacy at both individual and organisational levels.
Reflective and Lifelong Learning	Students acknowledged the importance of continued learning and reflection in managing polypharmacy effectively.	“This experience showed me that managing medications is an ongoing process, not a one-time decision.”	Encourages a commitment to lifelong learning in managing evolving medication challenges.

**Table 6:** Representative clinical scenarios illustrating student learning and deprescribing decisions.

Clinical Scenario (Patient and Comorbidities)	Medication Regimen	Identified Polypharmacy Issue	Student Interpretation and Response	Deprescribing Outcome and Rationale
Mrs. J, 82 years, dementia, hypertension, chronic pain	Donepezil, Ramipril, Amitriptyline, Diclofenac, Omeprazole	Cognitive impairment and frequent falls likely exacerbated by anticholinergic effects (Amitriptyline) and renal impact of NSAIDs (Diclofenac)	Students identified medications contributing to falls and confusion, recognising potential drug-induced cognitive impairment and risk of gastrointestinal and renal complications.	Discontinued Amitriptyline due to anticholinergic side effects and replaced Diclofenac with safer analgesics (e.g., paracetamol), significantly reducing fall risk.
Mr. A, 78 years, COPD, osteoporosis, type 2 diabetes	Salbutamol, Tiotropium, Alendronic Acid, Prednisolone, Metformin, Ibuprofen	Unnecessary long-term corticosteroid therapy causing hyperglycaemia and osteoporosis risk; Ibuprofen exacerbating gastrointestinal symptoms	Students correctly interpreted prolonged prednisolone as problematic for glycaemic control and bone density. Proposed safe corticosteroid tapering and recognised Ibuprofen as contraindicated due to gastrointestinal risks.	Initiated structured corticosteroid tapering; Ibuprofen replaced by topical analgesics to manage osteoarthritis pain safely.
Mrs. P, 84 years, atrial fibrillation, insomnia, anxiety	Warfarin, Atenolol, Diazepam, Zolpidem, Simvastatin	Sedative-hypnotic medications increasing risk of confusion, falls, and anticoagulation complications	Students identified diazepam and zolpidem contributing significantly to confusion and fall risk, proposing alternative non-pharmacological strategies for anxiety and sleep management.	Diazepam and Zolpidem gradually deprescribed; implemented behavioural interventions, reducing fall and confusion episodes.

## Data Availability

The data that support the findings of this study are available upon request from the corresponding author (W.J.).

## Appendix A. Student Evaluation Form

### Appendix A.1. Pre-Training Survey

Objective: To assess baseline knowledge, confidence, and attitudes toward polypharmacy management.

Instructions: Please rate the following statements on a scale of 1 to 5, where 1 = Strongly Disagree, 2 = Disagree, 3 = Neutral, 4 = Agree, 5 = Strongly Agree.

**Statement**	**1**	**2**	**3**	**4**	**5**
I feel confident identifying polypharmacy-related symptoms (e.g., fatigue, dizziness, confusion).					
I am familiar with the concept of polypharmacy as a chronic condition.					
I understand the role of prescribing cascades in medication-related problems.					
I am comfortable using tools like medication review checklists or electronic health records.					
I am aware of the importance of deprescribing as part of managing polypharmacy.					
I feel confident discussing medication-related issues with patients and caregivers.					

Open-Ended Questions:

1. What challenges do you foresee in identifying polypharmacy-related symptoms during patient interactions?
2. What do you hope to learn from this training?
3. How would you describe your current approach to medication review and deprescribing?

### Appendix A.2. Post-Training Survey

Objective: To evaluate changes in knowledge, confidence, and attitudes toward polypharmacy management.

Instructions: Please rate the following statements on a scale of 1 to 5, where 1 = Strongly Disagree, 2 = Disagree, 3 = Neutral, 4 = Agree, 5 = Strongly Agree.

**Statement**	**1**	**2**	**3**	**4**	**5**
I feel confident identifying polypharmacy-related symptoms (e.g., fatigue, dizziness, confusion).					
I am familiar with the concept of polypharmacy as a chronic condition.					
I understand the role of prescribing cascades in medication-related problems.					
I am comfortable using tools like medication review checklists or electronic health records.					
I am aware of the importance of deprescribing as part of managing polypharmacy.					
I feel confident discussing medication-related issues with patients and caregivers.					

Open-Ended Questions:

1. What was the most valuable skill or insight you gained from this training?
2. How has your approach to medication review and deprescribing changed after this experience?
3. What challenges do you still anticipate in managing polypharmacy, and how might you address them?

Reflective Journal Prompts

Objective: To encourage critical reflection on the experience of learning to manage polypharmacy.

Instructions: Use the following prompts to guide your reflective journal. Focus on specific insights, challenges, and strategies for future practice.

1. Describe a scenario where you identified polypharmacy as a primary cause of a patient’s symptoms. How did you address this issue?
2. Reflect on a situation where you struggled to propose a deprescribing strategy. What made it challenging, and how did you overcome it?
3. How has this training influenced your approach to patient-centred care and medication management?
4. What role did collaboration with pharmacists or other team members play in shaping your understanding of polypharmacy?
5. What strategies will you use to integrate polypharmacy management into your future clinical practice?

## Appendix B. Standardised Patient Feedback Form

Objective: To gather feedback on students’ communication and diagnostic performance during role-playing exercises.

Instructions: Please rate the following statements on a scale of 1 to 5, where 1 = Strongly Disagree, 2 = Disagree, 3 = Neutral, 4 = Agree, 5 = Strongly Agree.

**Statement**	**1**	**2**	**3**	**4**	**5**
The student demonstrated an understanding of how polypharmacy impacts my health and quality of life.					
The student communicated clearly and empathetically about medication-related concerns.					
The student identified potential issues with my medication regimen effectively.					
The student included me in the decision-making process regarding medication adjustments.					
The student’s approach made me feel heard and respected during the interaction.					

Open-Ended Questions:

1. What did the student do well during this interaction?
2. Was there anything the student could have done better? If so, please explain.
3. How did the student’s attention to medication-related issues impact your perception of the interaction?

## Appendix C. Sample Intervention Material

This appendix provides extensive representative examples of educational materials used in each phase of the intervention.

- Phase 1: Interactive Workshops

The first phase of the intervention involved interactive workshops, beginning with detailed case-based scenarios carefully crafted to illustrate polypharmacy-related clinical challenges. One illustrative scenario involved a simulated patient named Mr. Johnson, a 79-year-old male with hypertension, chronic obstructive pulmonary disease (COPD), type 2 diabetes, and osteoarthritis. Students analysed Mr. Johnson’s comprehensive medication list—including amlodipine, inhaled corticosteroids, metformin, ibuprofen, and tramadol—to identify potential adverse drug reactions and prescribing cascades.

Within these scenarios, students were guided to differentiate between disease progression and medication-induced symptoms. For instance, they explored how Mr. Johnson’s recent complaints of gastrointestinal discomfort and fatigue might relate to interactions between metformin and ibuprofen, potentially requiring deprescribing of ibuprofen to prevent renal impairment and gastrointestinal complications. Through active discussion, students learned to critically evaluate medication lists for safety and appropriateness.

Another significant component of this phase involved hands-on practice with Electronic Health Records (EHRs) and Clinical Decision Support Systems (CDSS). Students engaged directly with simulated patient records, practising the identification of problematic drug combinations, inappropriate medication dosages, and unnecessary prescriptions. An illustrative exercise involved evaluating medication records flagged for potential interactions, such as combinations of antihypertensives with non-steroidal anti-inflammatory drugs (NSAIDs).

Lastly, students participated in interactive group activities aimed at reinforcing their understanding of deprescribing principles. These activities included structured debates where students collaboratively weighed the risks and benefits of continuing versus discontinuing medications, enhancing their confidence and readiness to manage polypharmacy proactively.

- Phase 2: Practical Application

The practical application phase focused intensively on realistic simulated patient interactions. For example, students engaged with a detailed simulation involving Mrs. Garcia, an 84-year-old simulated patient presenting with confusion, dizziness, and repeated falls. Mrs. Garcia’s simulated medication regimen included benzodiazepines, diuretics, anticoagulants, statins, and analgesics. Students needed to determine whether these symptoms were a consequence of medication interactions or underlying medical conditions.

Following the initial simulated patient consultation, students undertook structured medication reviews in small groups, supervised by experienced pharmacists and clinical educators. They systematically assessed medication appropriateness, potential adverse effects, and opportunities for deprescribing. A notable scenario required students to identify that Mrs. Garcia’s falls and confusion could be related to benzodiazepine use, subsequently discussing alternative treatments to safely manage her anxiety and sleep disturbances.

A key component of this phase included the formulation of evidence-based deprescribing plans. Students practised communicating these plans clearly and empathetically to simulated patients, focusing on shared decision-making. The emphasis was on explaining the clinical reasoning behind deprescribing decisions and ensuring patient understanding and agreement.

Finally, each group of students participated in structured multidisciplinary team discussions, presenting their deprescribing recommendations to peers and educators. These discussions fostered interprofessional collaboration, allowed critical feedback, and refined the students’ clinical decision-making and communication skills.

- Phase 3: Reflection and Evaluation

The reflective phase involved comprehensive facilitated debriefing sessions designed to consolidate learning experiences. Educators guided students through reflective discussions, asking them to articulate their reasoning, describe challenges they faced, and reflect on their communication strategies with simulated patients. A common reflective prompt was: “Describe a scenario from your practical training where effective deprescribing significantly improved the simulated patient’s condition.”

In addition, students completed structured reflective journals. These journals encouraged detailed written reflections on their experiences, with prompts such as: “Reflect on an instance where you found it challenging to communicate deprescribing decisions to a simulated patient. How did you overcome these challenges, and what have you learned for future clinical interactions?” These exercises enabled students to deeply analyse their approach and develop strategies to address similar situations more effectively in future practice.

Furthermore, the reflective phase included reviewing recorded simulated patient interactions. Students critically evaluated their own performance, identifying areas of strength and opportunities for improvement, particularly focusing on clear communication of deprescribing rationale and patient-centred care. This reflective exercise significantly enhanced their self-awareness and self-directed learning.

Lastly, structured peer feedback sessions concluded this phase. Students presented reflections on their simulated patient encounters and received constructive feedback from their peers and educators. These feedback sessions emphasised collaborative learning, encouraged self-reflection, and reinforced the development of robust clinical reasoning and patient communication skills essential for managing polypharmacy.
